# Implicit theories and ability emotional intelligence

**DOI:** 10.3389/fpsyg.2015.00700

**Published:** 2015-05-22

**Authors:** Rosario Cabello, Pablo Fernández-Berrocal

**Affiliations:** ^1^Department of Psychology, Faculty of Education, University of Castilla-La Mancha, Ciudad Real, Spain; ^2^Department of Basic Psychology, Faculty of Psychology, University of Málaga, Málaga, Spain

**Keywords:** emotional intelligence, MSCEIT, implicit theories, gender, age, mediation

## Abstract

Previous research has shown that people differ in their implicit theories about the essential characteristics of intelligence and emotions. Some people believe these characteristics to be predetermined and immutable (entity theorists), whereas others believe that these characteristics can be changed through learning and behavior training (incremental theorists). The present study provides evidence that in healthy adults (*N* = 688), implicit beliefs about emotions and emotional intelligence (EI) may influence performance on the ability-based Mayer-Salovey-Caruso Emotional Intelligence Test (MSCEIT). Adults in our sample with incremental theories about emotions and EI scored higher on the MSCEIT than entity theorists, with implicit theories about EI showing a stronger relationship to scores than theories about emotions. Although our participants perceived both emotion and EI as malleable, they viewed emotions as more malleable than EI. Women and young adults in general were more likely to be incremental theorists than men and older adults. Furthermore, we found that emotion and EI theories mediated the relationship of gender and age with ability EI. Our findings suggest that people’s implicit theories about EI may influence their emotional abilities, which may have important consequences for personal and professional EI training.

## Introduction

Individuals can differ substantially in how much they believe that essential human characteristics such as intelligence and emotions are malleable. Those termed “entity theorists” regard such attributes as relatively fixed and difficult to change; others, termed “incremental theorists,” view the same attributes as malleable and changeable with effort and time (Dweck and Leggett, 1988). Being an incremental theorist has been associated with different positive outcomes such as better academic and professional achievement, and better mental health and well-being (Dweck, 2012), yet we are unaware of studies that have analyzed how an individual’s implicit theories of emotions and emotional intelligence (EI) influence his or her EI. The present study therefore sought to examine how such implicit theories relate to ability EI.

### Emotional Intelligence

In the quarter-century since Salovey and Mayer (1990) published the concept of EI, various theoretical models and instruments have been developed. The most widely applied theoretical models are mixed models and the ability model (Mayer et al., 2008). Mixed models conceptualize EI as a conglomeration of characteristics, including empathy, motivation, persistence, optimism, and social skills. Mixed EI is typically measured through self-report instruments, and it overlaps extensively with personality traits and measures of emotional/psychological well-being (Oconnor and Little, 2003; Grubb and McDaniel, 2007; Webb et al., 2013). The ability model, in contrast, defines EI as the integration of several capacities: “the ability to perceive accurately, appraise, and express emotion; the ability to access and/or generate feelings when they facilitate thought; the ability to understand emotion and emotional knowledge; and the ability to regulate emotions to promote emotional and intellectual growth” (Mayer and Salovey, 1997). Ability EI is assessed in adults using the Mayer-Salovey-Caruso Emotional Intelligence Test (MSCEIT; Mayer et al., 2002), which assesses each of the four abilities (“branches”) of perceiving, facilitating, understanding, and managing emotions (Mayer et al., 2003). MSCEIT scores show a positive relationship with various domains of daily life, including mental and physical health, social functioning, and academic and workplace performance (e.g., Ashkanasy and Daus, 2005; Daus and Ashkanasy, 2005; Mayer et al., 2008; Brackett et al., 2011; O’Boyle et al., 2011).

Ability EI appears to vary significantly with gender. While numerous studies of ability EI using such instruments as the MSCEIT have reported women to perform significantly better than men (e.g., Mayer et al., 1999; Ciarrochi et al., 2000; Brackett and Mayer, 2003; Palmer et al., 2005; Extremera et al., 2006; Goldenberg et al., 2006; Farrelly and Austin, 2007; McIntyre, 2010), the effect size of gender varies substantially. Some studies have reported small gender differences (e.g., Lumley et al., 2005; Fernández-Berrocal et al., 2012), while others have reported medium differences (e.g., Palmer et al., 2005; Farrelly and Austin, 2007). A meta-analysis of ability EI concluded the effect size to be 0.47 (Joseph and Newman, 2010).

Ability EI may also vary with age, but here the literature is less clear. While some studies reported significantly higher ability EI in older adults (Mayer et al., 1999), other studies have found no significant association between age and ability EI (Farrelly and Austin, 2007; Webb et al., 2013). Part of this discrepancy may arise from the fact that most studies on this question examine a relatively narrow age range in university populations, with all participants younger than 30 years. One study examining subjects ranging from 18 to 76 years old reported a small negative association between age and ability IE (Cabello et al., 2014).

### Implicit Theories

Implicit theories function like knowledge structures (Chiu et al., 1997; Plaks et al., 2009), through which people interpret themselves and others; individuals generally act in accordance with these theories (Dweck, 2012; Leith et al., 2014). Thus, implicit theories profoundly affect human behavior, and understanding natural variation in those theories may help predict how people will respond to particular stimuli, psychotherapy, or behavioral training. For example, individuals can hold different implicit theories about the malleability of various cognitive, emotional, and behavioral domains of human nature (Dweck et al., 1995a). These domains include intelligence, emotion, social skills, relationships, management skills, social judgment, and stereotyping (for a review, see Dweck, 2012). So-called entity theorists consider these domains as relatively fixed and difficult to change. In contrast, incremental theorists view these same attributes as malleable and changeable with effort and time (Dweck and Leggett, 1988).

Implicit theories of intelligence appear to be significant determinants of human psychology and behavior. Studies indicate that individuals with incremental theories of intelligence regard effort as positive and necessary for improving ability (Dweck and Leggett, 1988; Blackwell et al., 2007), and such people tend to set themselves learning goals aimed at enhancing their malleable traits (Dweck and Leggett, 1988; Robins and Pals, 2002; Mangels et al., 2006). They tend to be more persistent and strategic than those with entity theories of intelligence (Robins and Pals, 2002; Blackwell et al., 2007; Nussbaum and Dweck, 2008), and they are more likely to cultivate mastery-oriented strategies to overcome developmental challenges such as the start of adolescence and the transition from primary school to secondary school (Blackwell et al., 2007).

Implicit theories of emotions also strongly affect human psychology and behavior. Several studies with university students or adults have shown that compared to those holding entity theories about the malleability of emotions, those holding incremental theories more frequently use cognitive reappraisal as an emotional regulation strategy, experience more positive and fewer negative emotions, receive greater social support, are more likely to use mastery-oriented strategies rather than helpless strategies, and harbor higher expectations of success (Tamir et al., 2007; Burnette et al., 2013; De Castella et al., 2013).

Schumann et al. (2014) reported that people’s beliefs about empathy are positively associated with empathic concern (Study 1, *r* = 0.21), based on a measure of dispositional tendencies to feel empathy for others in everyday life. In fact, data from that study suggest that empathic behavior is more strongly associated with people’s beliefs about empathy than with their self-reported levels of empathy. This suggests that implicit theories of empathy and self-reported levels of empathy are related but distinct, which coincides with a meta-analysis of self-report studies (Burnette et al., 2013), indicating only a small to moderate relationship between implicit theories and self-regulatory processes (range, 0.15–0.24). Thus, the available evidence suggests that people’s theories about whether or not emotional abilities can change and be developed does not overlap with—and can be separated from—what people self-report about their emotional abilities.

Similarly, implicit theories of intelligence and emotions are related but distinct; some academic and emotional outcomes are affected by both theories, while others are affected only by one or the other (Romero et al., 2014). In addition, several studies with university students suggest that they view emotions as more malleable than intelligence (Tamir et al., 2007).

Whether age and gender affect implicit theories about intelligence and emotions is unclear. Some studies have failed to show significant differences between men and women (Dweck et al., 1995a; Tamir et al., 2007; Doron et al., 2009), and a meta-analysis found the available evidence to be inconclusive (Burnette et al., 2013). Similarly, while some studies have found no significant association between age and implicit theories (Dweck et al., 1995a), others have reported that people differentiate into incremental and entity theorists around 13 years of age (Diseth et al., 2014), and that older adults (ranging from 18 to 70 years) are more likely to hold entity theories (Spinath et al., 2003).

### The Present Study

Although implicit theories have been linked with numerous domains of human nature, we are unaware of studies exploring possible relationships of theories about emotions or EI with ability EI. This led to the present study with the following objectives (O) and hypotheses (H):

O1. Examine how implicit theories of emotions and EI are related and how much individuals perceive these domains to be malleable. We hypothesized that emotions would be perceived as more malleable than EI (H1), based on previous work (Tamir et al., 2007).
O2. Examine whether and how implicit theories of emotion and EI are associated with ability EI. Because implicit theories have domain-specific implications (Dweck et al., 1995a; Romero et al., 2014), we expected implicit theories of EI to be more linked to ability EI and more predictive of ability EI than implicit theories of emotion (H2).
O3. Examine possible effects of gender and age on implicit theories about emotions and EI.
O4. Determine whether implicit theories about emotions and EI mediate the relationship of gender or age with ability EI.

## Materials and Methods

### Participants and Procedure

The sample comprised 688 healthy adults (37% men) ranging in age from 18 to 73 years (*M* = 36.02, SD = 14.9). Participants were recruited via posters on the local university campus, at retirement homes and in local newspapers. They received no financial compensation or incentive in exchange for participation in the study. Participants were accepted into the study to ensure a broad, balanced distribution of gender, age, and socio-economic status. Volunteers were not accepted into the study if they were younger than 18 years or if they had any physical or psychological disability that would compromise their ability to fill out the MSCEIT. Participants completed the questionnaires themselves at home. Data were collected over two consecutive years with the help of a team of research assistants. The study was carried out in accordance with the Declaration of Helsinki. Ethics approval was obtained from the Research Ethics Committee, University of Málaga.

### Instruments

*Mayer-Salovey-Caruso Emotional Intelligence Test* (Version 2.0; Mayer et al., 2002; Extremera and Fernández-Berrocal, 2009). Ability EI was measured using a Spanish translation of the MSCEIT, which shows similar psychometric properties as the original instrument (Extremera et al., 2006); this test has been validated for adults aged 17 and older. The MSCEIT uses two tasks to measure each of the four branches of EI (perceiving, facilitating, understanding, and managing emotions), comprising a total of eight tasks. The instrument provides separate scores for each branch as well as an overall score for total EI. Scores can be calculated based on expert or consensus norms, which correlate strongly with each other (*r* > 0.90; Mayer et al., 2003). In the present study, we used only total EI score, which we calculated using consensus norms. Scores computed by the test publishers are standardized (*M* = 100, SD = 15), and the reliability of the two halves is 0.93 based on the consensus criterion. The test-retest reliability of the global MSCEIT is 0.86 after 3 weeks (Brackett and Mayer, 2003).

*Implicit Theories of Emotion* (Tamir et al., 2007). We evaluated implicit theories about the malleability of emotions using the scale adapted by Tamir et al. (2007) from the scale originally created by Dweck (1999) to evaluate implicit theories about intelligence. The scale consists of four items, two assessing *incremental theories* (“Everyone can learn to control their emotions”; “If they want to, people can change the emotions that they have”) and two assessing *entity theories* (“No matter how hard they try, people can’t really change the emotions that they have”; “The truth is, people have very little control over their emotions”). The participants were asked to report their agreement on a 7-point Likert scale. Entity statements were reverse-scored such that higher scores reflected a more incremental theory of emotions. The Spanish translation of the *Implicit Theories of Emotion* instrument was created using a back-translation procedure involving two independent translators, both of whom have PhDs in psychology and who are experts in the topic. In the present sample, the Cronbach alpha was 0.75.

*Implicit Theories of EI*. Implicit theories about the malleability of EI were evaluated using a scale adapted by us from the scale originally developed by Dweck (1999) to evaluate implicit theories about intelligence. The scale comprises two statements assessing *incremental theories* (“No matter how much emotional intelligence you have, you can always change it quite a bit”; “You can always significantly change how emotionally intelligent you are”) and two assessing *entity theories* (“You can learn new things, but you can’t really change your basic emotional intelligence”; “Your emotional intelligence is something about you that you can’t change very much”). Participants were asked to report their agreement on a 7-point Likert scale. Entity statements were reverse-scored such that higher scores reflected a more incremental theory of emotions. The Spanish translation of the *Implicit Theories of Emotional Intelligence* instrument was created using a back-translation procedure involving two independent translators, both of whom have PhDs in psychology and who are experts in the topic. In the present sample, the Cronbach alpha was 0.74.

### Statistical Analysis

All statistical analyses were carried out using SPSS 20.0 (IBM, USA). Preliminary analyses were carried out to compute descriptive statistics, as well as to detect relationships among age, gender, implicit theories about emotions or EI, and total ability EI scores. To examine whether theories about emotions or about EI explained a greater proportion of the observed variance in ability EI, we conducted two analyses of two-step hierarchical regression in which the predictor variables were implicit theories of emotions or EI, while the criterion variable was total ability EI score. To determine whether gender and age were related to ability EI through implicit theories about emotions or EI, we used a parallel mediation model in the PROCESS algorithm (Hayes, 2013). PROCESS is a conditional process modeling program for use with SPSS that utilizes an ordinary least squares- or logistic-based path analytical framework to test for both direct and indirect effects (Hayes, 2013). To test the significance of a mediation effect, PROCESS generates the Sobel test value and percentile-based bias-corrected bootstrap confidence intervals (CIs) for each indirect effect; the indirect effect is judged significant when the CI does not contain 0 (*p* < 0.05).

## Results

### Preliminary Analyses

Means, standard deviations, and correlations for all variables are shown in Table 1. Our sample tended to view as malleable both emotions (*M* = 19.69) and EI (*M* = 18.92; Table 1). This is consistent with previous studies on implicit theories of emotions (Tamir et al., 2007) and intelligence (Dweck et al., 1995a). In addition, our subjects viewed emotions as more malleable than EI based on a paired-samples *t* test [*t*(687) = 4.20, *p* < 0.0001]. This supports our hypothesis H1 and is consistent with previous studies on implicit theories of emotions (Tamir et al., 2007) and intelligence (Dweck et al., 1995a).

**TABLE 1:** **Descriptive statistics and correlations**.

****	***M***	**SD**	**1**	**2**	**3**	**4**	**5**
1. Gender	0.63	0.48	–				
2. Age	36.02	14.87	–0.23**	–			
3. Theories of EI	18.92	4.33	0.22**	–0.35**	–		
4. Theories of emotions	19.69	4.61	0.19**	–0.27**	0.43**	–	
5. Ability EI	102.62	12.63	0.19**	–0.30**	0.26**	0.21**	–

*p < 0.05; **p < 0.01.

Implicit theories of emotions and EI significantly and positively correlated with each other with a medium effect size (*r* = 0.43, *p* < 0.01). Thus, both types of theory were related but distinct and separable in our study. We performed all subsequent analyses using both implicit theories.

### Implicit Theories of Emotions and Total Ability EI

Implicit theories of both emotion and EI correlated significantly with ability EI scores on the MSCEIT. Incremental theories were associated with higher ability EI. To determine whether theories of emotions or EI explained a greater proportion of the observed variance in ability EI, we conducted two analyses of two-step hierarchical regression to isolate the variance explained by each scale. The predictor variables were implicit theories of emotions or EI, while the criterion variable was total ability EI score. In the *first model*, we performed regression by adding implicit theories of emotions to the model, followed by implicit theories of EI. In the *second model*, the order of addition of the implicit theories to the model was reversed to control for the variance explained by implicit theories of EI. Both types of implicit theories explained a relatively small proportion of the variance in total ability EI. Higher scores in either type of implicit theory predicted higher total ability EI. In the *second model*, when implicit theories of EI were added first to the model, a larger proportion of variance was explained (*R*^2^ = 0.07; Table 2). In other words, theories of EI explained a larger proportion of the total variance.

**TABLE 2:** **Regression to predict total ability EI from implicit theories of either emotion or EI**.

**Criterion variable**	**β**	
	**Step**	**Final**
Ability EI		
Model 1		
Theories of emotions	0.21**	0.12*
Theories of EI		0.21**
*R*^2^	0.04**	0.08**
*R*^2^ change		0.04**
Model 2		
Theories of EI	0.26**	0.21**
Theories of emotions		0.12*
*R*^2^	0.07**	0.08**
R^2^ change		0.01*

*p < 0.05; **p < 0.01.

### Gender and Age Differences in Implicit Theories of Emotion or EI

In order to investigate O3, we analyzed whether gender and age were related to implicit theories of emotions and EI. Women held significantly more incremental theories of emotions than men [*t*(432) = –4.99, *p* < 0.0001, *d* = 0.36] with a small effect size, and significantly more incremental theories of EI [*t*(432) = –5.91, *p* < 0.0001, *d* = 0.50] with a medium effect size. At the same time, women showed significantly higher total ability EI [*t*(432) = –4.88, *p* < 0.0001, *d* = 0.35] with a small effect size. Incremental theories of emotions and EI correlated negatively with age: older adults were less likely than younger ones to hold incremental theories. Age, in turn, correlated negatively with ability EI: older people scored lower for total ability EI than younger adults.

### Mediation by Implicit Theories of the Association of Age or Gender with Total Ability EI

We used a parallel mediation model to explore whether gender and age were related to total ability EI via implicit theories of emotions and EI (O4). We used PROCESS Model 4 for parallel mediation, and all indirect effects were subjected to follow-up bootstrap analyses with 10,000 bootstrap samples and 95% bias-corrected CI. In this way, predictor variables (gender and age), the criterion variable (ability EI) and mediators (implicit theories of emotions and EI) were tested in a single model.

**FIGURE 1:**
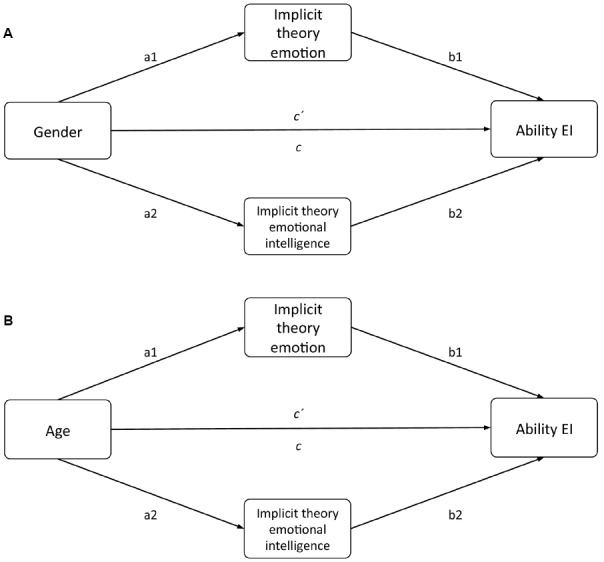
**Hypothesized parallel mediation model**.

In Table 3, column *a* shows the effect of gender or age on the mediators (implicit theories of emotions and EI); column *b*, the effect of implicit theories on the criterion variable (ability EI); column *c*, the total effect (direct and indirect) of gender or age on total ability EI; and column *c*′, the direct effect of gender or age on total ability EI while controlling implicit theories. The results show that both types of implicit theories mediated the relationship of gender and age with total ability EI (Figure 1). The bootstrapped 95% CI confirmed that both types of implicit theories exerted significant indirect effects on the relationship of gender or age with total ability EI. When the combination of gender and both types of implicit theories was examined, gender accounted for 3.4% of the observed variance in total ability EI; both types of implicit theories accounted for 6% (overall *R*^2^ = 0.094). When the combination of age and both types of implicit theories was examined, age accounted for 9% of the observed variance in total ability EI; both types of implicit theories accounted for 3% (overall *R*^2^ = 0.12).

**TABLE 3:** **Implicit theories of emotions or EI as mediators of the relationship of gender or age with total ability EI**.

**Predictor**	**Criterion**	**Mediator**	***a***	***b***	***c***	***c*′**	**Sobel *z***	95% CI
Gender	Ability EI	Theories of emotions	1.804** (0.357)	0.285* (0.111)	4.895** (0.979)	3.293** (0.981)	2.257*	[0.146, 1.043]
		Theories of EI	1.997** (0.333)	0.544** (0.119)	4.895** (0.979)	3.293** (0.981)	3.602**	[0.577, 1.770]
Age	Ability EI	Theories of emotions	–0.083** (0.011)	0.238* (0.110)	–0.254** (0.031)	–0.191* (0.033)	2.056*	[–0.034, –0.003]
		Theories of EI	–0.102** (0.010)	0.418** (0.120)	–0.254** (0.031)	–0.191* (0.033)	–3.264**	[–0.070, –0.019]

*Standard errors are presented in parentheses below the non-standardized B coefficients. Column* a *shows the coefficient of gender or age in the regression to predict the mediator; column *b*, the coefficient of the mediator in the regression to predict the criterion variable; column* c*, the coefficient of gender or age in the regression to predict the criterion variable; and column* c′, the coefficient of gender or age in the regression to predict the criterion variable while controlling the mediator. *p < 0.05; **p < 0.01.

## Discussion

This study is the first to our knowledge that provides evidence that implicit theories of emotion and EI influence total ability EI on the MSCEIT, such that incremental theorists show higher ability EI. This finding is consistent with hypothesis H2 and with previous studies showing that those who believe they can change their emotions use effective strategies such as cognitive reappraisal and mastery-oriented strategies more frequently than individuals who hold entity theories, and that incremental theorists experience fewer negative emotions (Tamir et al., 2007; Burnette et al., 2013). Our finding is also consistent with studies showing that university students holding incremental theories about intelligence adopt mastery-oriented strategies to face developmental challenges such as the start of adolescence and the change from primary school to high school (Blackwell et al., 2007; Cury et al., 2008).

Our participants perceived implicit theories about both emotion and EI as malleable, and they viewed theories about emotions as more malleable, consistent with our hypothesis H1. This result suggests that people tend to think of emotions as less stable than EI, consistent with previous work on implicit theories of emotions and intelligence (Tamir et al., 2007), as well as with widespread notions of the stability of intelligence (Spinath et al., 2003).

Our data further indicate that implicit theories about emotions and EI, while related, do not overlap and can be separated from each other. A similar relationship has been reported for implicit theories of emotion and intelligence (Tamir et al., 2007), such as when predicting academic and emotional functioning in adolescents (Romero et al., 2014). Our data suggest that one key difference between theories of emotions and of EI is that they appear to influence total ability EI to different extents. We found that theories of EI explained more of the observed variance in ability EI than theories of emotions, consistent with our hypothesis H2.

Motivated in part by the lack of clear evidence for or against an association of gender or age with implicit theories of emotions or intelligence, we wanted to examine whether either sociodemographic variable was associated with incremental or entity theories in our sample (O3). We found that women had more incremental theories of emotions and EI than men, in accordance with findings from Spinath et al. (2003), but in contrast to several studies reporting no significant difference between genders (Dweck et al., 1995a; Tamir et al., 2007; Doron et al., 2009). This discrepancy may reflect the fact that many previous studies used relatively young, university-based samples that may not be representative of the general adult population. In the present study, we analyzed a sample of 688 adults with ages ranging from 18 to 73 years; therefore, our results may provide a more valid point of reference than previous studies. We found that young adults were more likely than older ones to have incremental theories, which is consistent with previous work (Spinath et al., 2003). These findings suggest that women and young adults are more likely to hold incremental theories than men and older adults.

We found another significant effect of gender and age: women and young adults in our sample showed higher total ability EI than men or older individuals. Similar results have been reported in previous work (Joseph and Newman, 2010; Fernández-Berrocal et al., 2012; Cabello et al., 2014). This result, together with our finding that gender and age influence implicit theories of emotions and EI, led us to identify both types of theories as mediators of the relationship of gender and age with total ability EI (O4). This suggests that implicit theories may help to explain the observed influence of gender and age on ability EI. Even so, implicit theories explained only 6% of the observed variance in ability EI, while gender explained only 3.4%. The fact that most variance was not explained by the factors we examined indicates the existence of other, more important factors, which should be examined in future studies. Our findings serve as a cautionary tale against simplistic interpretations of emotion or EI as being determined primarily by gender or age.

Our results associating incremental theories about EI with higher total ability EI extends previous studies linking incremental theories with better academic achievement, more effective decision-making, and more effortful behavior (Dweck, 1999; Blackwell et al., 2007). This raises the question of how implicit theories of EI may influence ability EI. It stands to reason, based on Dweck’s early research on implicit theories, that people who believe that EI can be cultivated will be more willing to make efforts to do so through hard work, appropriate strategies and seeking help from others. Such individuals may be more likely to view an emotionally or socially challenging situation not as a potential danger but as an opportunity for emotional learning and progress. Future studies should examine whether and how implicit theories of EI and ability EI are associated with such real-life consequences.

These findings may have important implications for interventions with adults. For example, it may be advisable to complement EI interventions with guided reflection or writing tasks about the malleability of emotions and EI. This may help ensure that the subsequent EI intervention has a profound and long-lasting effect. Previous work has shown that direct and indirect interventions in adolescents and adults can increase incremental beliefs about intelligence, leading to long-lasting effects (Aronson et al., 2002; Blackwell et al., 2007). Indeed, training in the malleability of several human domains, such as emotions, has been shown to be useful not only for improving academic and professional performance but also for learning and developing (Dweck et al., 1995b; Dweck, 2012; Burnette et al., 2013). Our data suggest that men and older people may benefit in particular from interventions that target implicit theories of emotions and EI, since these individuals are more likely to hold entity theories about these domains. Developing their incremental beliefs may facilitate improvement in ability EI. It may be more efficient to target implicit theories of EI than theories of emotions, since most individuals in our sample considered EI to be less malleable. Future studies should explore effective methods for integrating interventions to increase incremental beliefs about EI into training programs. For example, studies should examine whether interventions to consolidate and support EI change are more effective when conducted at the beginning or end of EI-based training.

### Limitations and Future Directions

While this study does provide the first evidence linking implicit theories of emotions and EI with ability EI, its conclusions are limited in several ways. Its cross-sectional nature and reliance on a single instrument to assess ability EI preclude inferences about causality in the relationship between implicit theories and EI. Our research was inspired by Dweck’s implicit theory and previous findings suggesting that implicit theories predict distinct emotional abilities (Burnette et al., 2013; Schumann et al., 2014). However, our cross-sectional design prevents us from excluding the possibility of causality in the opposite direction: people with high EI may gradually develop more incremental theories than people with low EI. Prospective studies are needed to verify our mediation results suggesting that incremental theories lead to higher ability EI. It would be quite interesting to ask, for example, whether manipulating implicit theories of emotions or EI durably increases EI functioning. Longitudinal interventional studies should also test whether training that addresses implicit theories of EI can lead to better mental and social functioning than EI training alone. Another limitation of our study is that we examined only implicit theories of emotions and EI but not several other variables, such as implicit theories of intelligence, that may also contribute to the observed variance in total ability EI. Indeed, the variables in our model explain only a small proportion of the variance, suggesting the existence of unidentified factors.

Future work along these lines may provide more insight into how the association between implicit theories and EI evolves over a lifetime. Evidence suggests that training in social and emotional competencies is crucial and should begin in early years and continue throughout one’s lifetime (Ruiz-Aranda et al., 2012; Rivers et al., 2013; Kumschick et al., 2014). Future studies should also examine the relationships between implicit theories of EI and self-report measures of EI to examine whether they are related to each other similarly or differently to how they are related to ability EI.

In summary, our findings contribute to the growing literature on implicit theories and emotional functioning, which affects quality of life, social adjustment, and professional performance. Incremental theories about intelligence and emotions have already been shown to contribute to an orientation toward learning and development (Dweck, 2012; Yeager and Dweck, 2012). Here we illustrate how implicit theories about emotions and EI are associated with ability EI, softening the impact that socio-demographic factors such as gender and age may have. This suggests that manipulating those theories may be useful in EI training.

### Conflict of Interest Statement

The authors declare that the research was conducted in the absence of any commercial or financial relationships that could be construed as a potential conflict of interest.
